# Subconjunctival injection of mesenchymal stem cells for corneal failure due to limbal stem cell deficiency: state of the art

**DOI:** 10.1186/s13287-020-02129-0

**Published:** 2021-01-13

**Authors:** Sara Galindo, Ana de la Mata, Marina López-Paniagua, Jose M. Herreras, Inmaculada Pérez, Margarita Calonge, Teresa Nieto-Miguel

**Affiliations:** 1 Instituto de Oftalmobiología Aplicada (IOBA), Universidad de Valladolid, Edificio IOBA, Campus Miguel Delibes, Paseo de Belén 17, 47011 Valladolid, Spain; 2 Centro de Investigación Biomédica en Red de Bioingeniería, Biomateriales y Nanomedicina (CIBER-BBN), Instituto de Salud Carlos III, Madrid, Spain; 3 Centro en Red de Medicina Regenerativa y Terapia Celular de Castilla y León, Valladolid, Spain

**Keywords:** Subconjunctival injection, Cornea, Limbus, Regeneration, Mesenchymal stem cells, Limbal stem cell deficiency, Corneal epithelium

## Abstract

Mesenchymal stem cells (MSCs) have unique and beneficial properties and are currently used to treat a broad variety of diseases. These properties include the potential for differentiation into other cell types, secretion of different trophic factors that promote a regenerative microenvironment, anti-inflammatory actions, selective migration to damaged tissues, and non-immunogenicity. MSCs are effective for the treatment of ocular surface diseases such as dry eye, corneal burns, and limbal stem cell deficiency (LSCD), both in experimental models and in humans. LSCD is a pathological condition in which damage occurs to the limbal epithelial stem cells, or their niche, that are responsible for the continuous regeneration of the corneal epithelium. If LSCD is extensive and/or severe, it usually causes corneal epithelial defects, ulceration, and conjunctival overgrowth of the cornea. These changes can result in neovascularization and corneal opacity, severe inflammation, pain, and visual loss. The effectiveness of MSCs to reduce corneal opacity, neovascularization, and inflammation has been widely studied in different experimental models of LSCD and in some clinical trials; however, the methodological disparity used in the different studies makes it hard to compare outcomes among them. In this regard, the MSC route of administration used to treat LSCD and other ocular surface diseases is an important factor. It should be efficient, minimally invasive, and safe. So far, intravenous and intraperitoneal injections, topical administration, and MSC transplantation using carrier substrata like amniotic membrane (AM), fibrin, or synthetic biopolymers have been the most commonly used administration routes in experimental models. However, systemic administration carries the risk of potential side effects and transplantation requires surgical procedures that could complicate the process. Alternatively, subconjunctival injection is a minimally invasive and straightforward technique frequently used in ophthalmology. It enables performance of local treatments using high cell doses. In this review, we provide an overview of the current status of MSC administration by subconjunctival injection, analyzing the convenience, safety, and efficacy for treatment of corneal failure due to LSCD in different experimental models. We also provide a summary of the clinical trials that have been completed, are in progress, or being planned.

## Background

Corneal damage is one of the main causes of blindness. To safeguard the visual function, it is necessary to preserve corneal transparency, which depends on many factors. One of the critical aspects of corneal transparency is the health of the epithelial barrier, which must be constantly renewed to accomplish its many vital functions. This continuous epithelial turnover is possible because of a population of limbal epithelial stem cells (LESCs) located at the basal layer of the corneoscleral limbal niche [1–4]. Destruction or dysfunction of the LESCs or their niche induces limbal stem cell deficiency (LSCD). LSCD syndrome is characterized by the presence of an unstable epithelium with subsequent ulceration, ingrowth of conjunctival tissue onto the corneal epithelium, neovascularization of the corneal surface, and persistent inflammation and chronic pain, all of which can ultimately cause vision loss due to corneal opacity [5].

Cultivated limbal epithelial transplantation is the current treatment of choice for treating patients suffering from ocular surface failure due to LSCD [6]. Although it represents one of the first and most recognizable successes of regenerative medicine, this treatment is not exempt from limitations, such as the low availability of donors and limited success in the most severe cases [7–10].

Mesenchymal stem cells (MSCs) are considered to be a very attractive candidate for cell-based therapies in several clinical applications. There are already numerous works indicating that the therapeutic effects of MSCs rely not only on their innate differentiation capacity, but also on their immunomodulatory and anti-inflammatory properties to repair damaged tissues [11]. MSCs have been widely studied as a successful therapy to treat ocular surface failure due to LSCD. They facilitate recovery of the corneal epithelium and reduce corneal opacity and inflammation of the ocular surface, not only in experimental models but also in humans [12].

Currently, there is a lack of consensus regarding the best route to administer MSCs to the ocular surface for corneal regeneration. Subconjunctival injection, the focus of the present review, is a straightforward technique that is frequently used in the daily ophthalmologic practice to administer different drugs. This approach employs a simple, safe, and minimally invasive technique to deliver locally high cell doses in a low volume [13].

Besides, there are different techniques that have been commonly used so far. In some preclinical studies, MSCs were administered topically [14–16], using natural or synthetic substrata such as amniotic membrane (AM) [17–21], fibrin [22], or films made of poly-L-lactic acid [14, 23, 24] or polyamide [25]. In other studies, the cells were injected intravenously [26–28], intraperitoneally [29, 30], intracorneostromally [31, 32], or subconjunctivally [33–39]. There are also human clinical studies in which AM [12], sub-tenon injections (*clinicaltrials.gov_NCT04224207, NCT02144103*, and *NCT03011541*), or subconjunctival injections are used to administer the MSCs (*clinicaltrials.gov_NCT02325843, NCT01808378, NCT04484402, NCT03967275*, and *NCT03237442*). The first clinical trial performed and published using bone marrow (BM)-MSCs on AMs (*clinicaltrials.gov_NCT01562002*) was demonstrated to be both safe and effective in the restoration of the corneal epithelial phenotype for the treatment and improvement of patients suffering from LSCD [12]. Additionally, sub-tenon injection is also a suitable ocular route of drug administration that involves the delivery of medication or cells through the area between the sclera and the Tenon capsule. For instance, injection of umbilical cord Wharton’s jelly-derived MSCs into the sub-tenon space had beneficial effects on visual functions in retinitis pigmentosa patients by reactivating the degenerated photoreceptors (*clinicaltrials.gov_NCT04224207*) [40]. In addition, two more clinical trials using sub-tenon injection to transplant adipose tisue (AT)-MSCs (*clinicaltrials.gov_NCT02144103*) and BM-MSCs (*clinicaltrials.gov_NCT03011541*) are in progress for the treatment of glaucomatous neurodegeneration and retinal and optic nerve damage, respectively.

However, there are some drawbacks to these administration routes that are not present with subconjunctival injection: (1) systemic administration presents a high risk of side effects, and the number of cells that reach the target tissue is low; (2) topical administration involves loss of cells, as they are not retained on the ocular surface for a long time; and (3) the use of carrier substrata requires a surgical procedure, increasing the cost, while limiting the number of cells that can be transplanted.

In this review, we provide an overview of the current status of MSC administration by subconjunctival injection, analyzing its convenience, safety, and efficacy for the treatment of corneal failure due to LSCD. We also identify clinical trials that are completed, in progress, or that are planned.

## Main text

### Use of MSCs for treating corneal epithelial damage

MSCs constitute an adult stromal stem cell population that originates from the mesoderm. Although BM and AT are the most utilized sources, MSCs are also present in muscle, cartilage, dental pulp, umbilical cord, placenta, and in the limbal stroma of mammalian eyes, including humans [11, 41]. To standardize the characterization of these cells, the International Society for Cellular Therapy established three minimal criteria [42]: (1) adherence to plastic surfaces in standard culture conditions; (2) multipotent differentiation potential to form bone, cartilage, and adipose cells in vitro; and (3) presentation of a specific surface-antigen expression pattern, including CD90, CD105, and CD73, but without CD34, CD45, CD11b or CD14, CD19 or CD79α, and HLA-DR [42].

Several in vitro and in vivo studies using MSCs for corneal epithelium regeneration have been published recently. All of these works, performed in different animal models, present encouraging results regarding safety, corneal epithelium regeneration, transparency recovery, healing process, and ultimately vision restoration [17, 23, 26, 43]. These results could be due either to the transdifferentiation of the transplanted MSCs into corneal epithelial cells or to other well-known features of MSCs, such as migration towards the injured areas, secretion of trophic and growth factors capable of stimulating resident stem cells, and reducing tissue injury and inflammation [23, 26, 44, 45].

MSC-secreted growth factors are considered essential for the proliferation and migration of corneal epithelial cells, and they contribute to the corneal epithelium regenerative process [46–49]. The anti-inflammatory action of MSCs is associated with secreted soluble factors [29, 35] that suppress the infiltration of inflammatory cells and CD68+ macrophages in the damaged tissue, inhibiting the expression of inflammatory proteins [16, 33, 35].

MSCs have reduced expression of major histocompatibility complex (MHC) class I antigens, and they do not express MHC II or co-stimulatory molecules like CD80, CD86, and CD40 [50, 51]. Thus, the MSCs have a non-immunogenic phenotype, making it possible to use them allogeneically in cornea regeneration and avoiding the need of immunosuppression after transplantation.

### MSC administration routes for treating corneal epithelial damage

The route of MSC delivery is one of the main problems to overcome in achieving optimal benefits from stem cell therapy. While some authors, using either intravenous or intraperitoneal systemic administration of MSCs, reported the migration of stem cells to the injured cornea [26, 28, 52], others did not [29], suggesting instead that the therapeutic effect was due to the trophic factors secreted by MSCs. Some studies indicate that intravenous administration during the acute phase of corneal epithelial damage can improve clinical signs such as epithelial defects, neovascularization, and corneal opacity, in both mice [26, 28] and rabbits [27].

The use of cell carriers for MSC transplantation is one of the most frequently applied techniques. Clinical signs are reduced when MSCs are transplanted to the ocular surface using AM as carrier both in experimental models [17–21] and in humans [12]. However, the cell dose that can be delivered is normally lower than by using other routes. In addition, as a human product, AM has limited availability, risk of disease transmission, and a high economic cost [53]. Another natural carrier is fibrin gel, capable of helping repair the ocular surface when transplanted with or without MSCs [22]. Moreover, synthetic cell carriers such as contact lenses [54], poly-L-lactic acid [14, 23], and polyamide [25] have been studied to find reproducible substrata that allow cell adhesion, viability, proliferation, and regeneration of the ocular surface when they are transplanted with MSCs. However, the number of stem cells delivered by these carriers is limited compared to the cell dose that can be administered by injections. Additionally, most of the substrata require suturing to the ocular surface. This extra step in the surgical intervention and the follow-up surgery to remove the stitches make the whole process more tedious, risky, and expensive.

In contrast to the other delivery protocols, topical administration of MSCs has been used in LSCD experimental models [14, 15]. While clearly simpler than the delivery methods described above, it has some drawbacks such as low retention time on the ocular surface, high washing rate, and low permeability of the corneal epithelium.

**Fig. 1:**
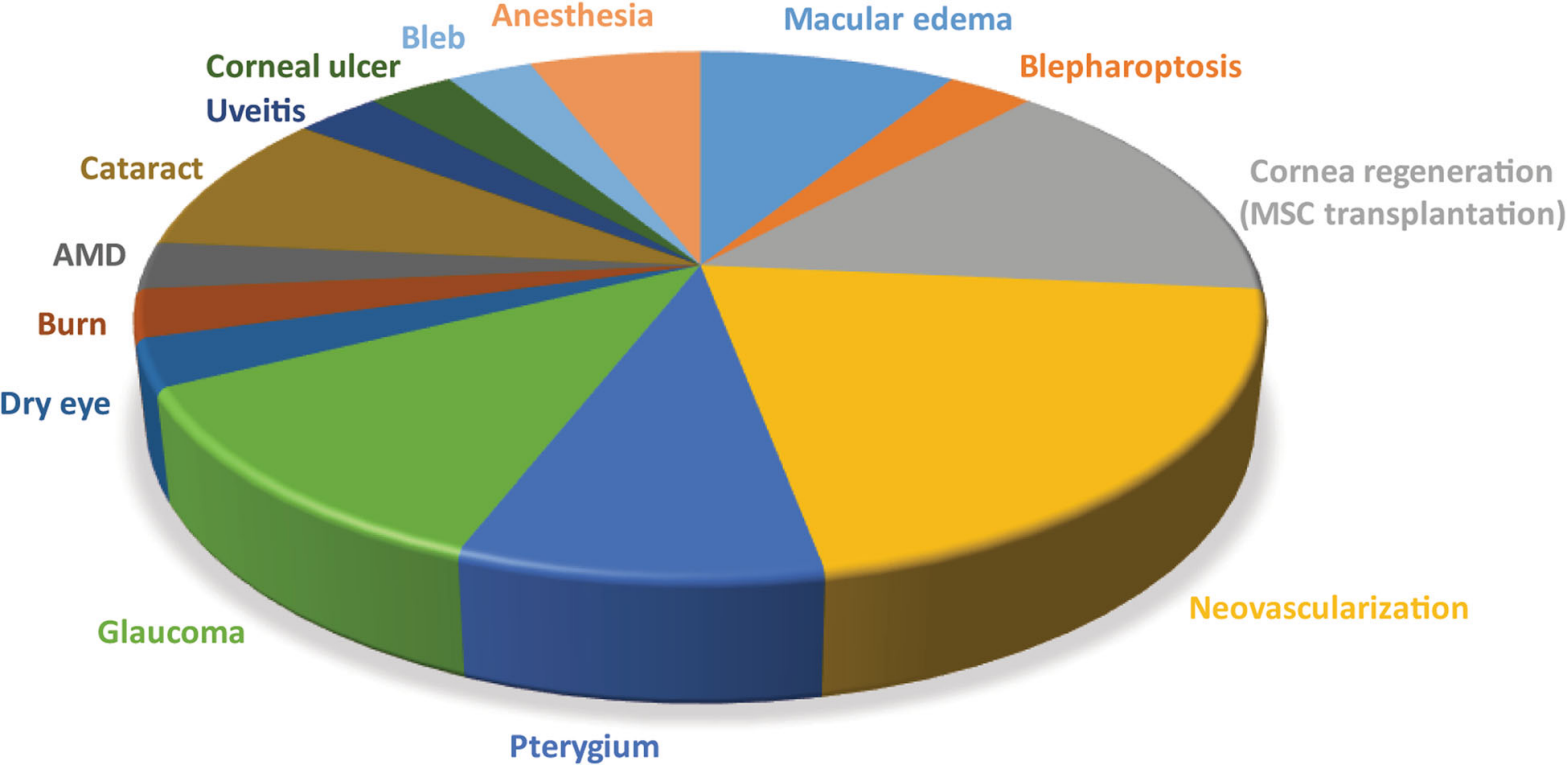
Pie chart depicting the current application of subconjunctival injections in clinical trials (*clinicaltrials.gov*). Macular edema (9%), blepharoptosis (3%), cornea regeneration (MSC transplantation) (14%), neovascularization (20%), pterygium (9%), glaucoma (12%), dry eye (3%), burn (use of vitamin C) (3%), age-related macular degeneration (AMD) (3%), cataract (9%), uveitis (3%), keratitis (3%), bleb (3%), and anesthesia (6%)

**Fig. 2:**
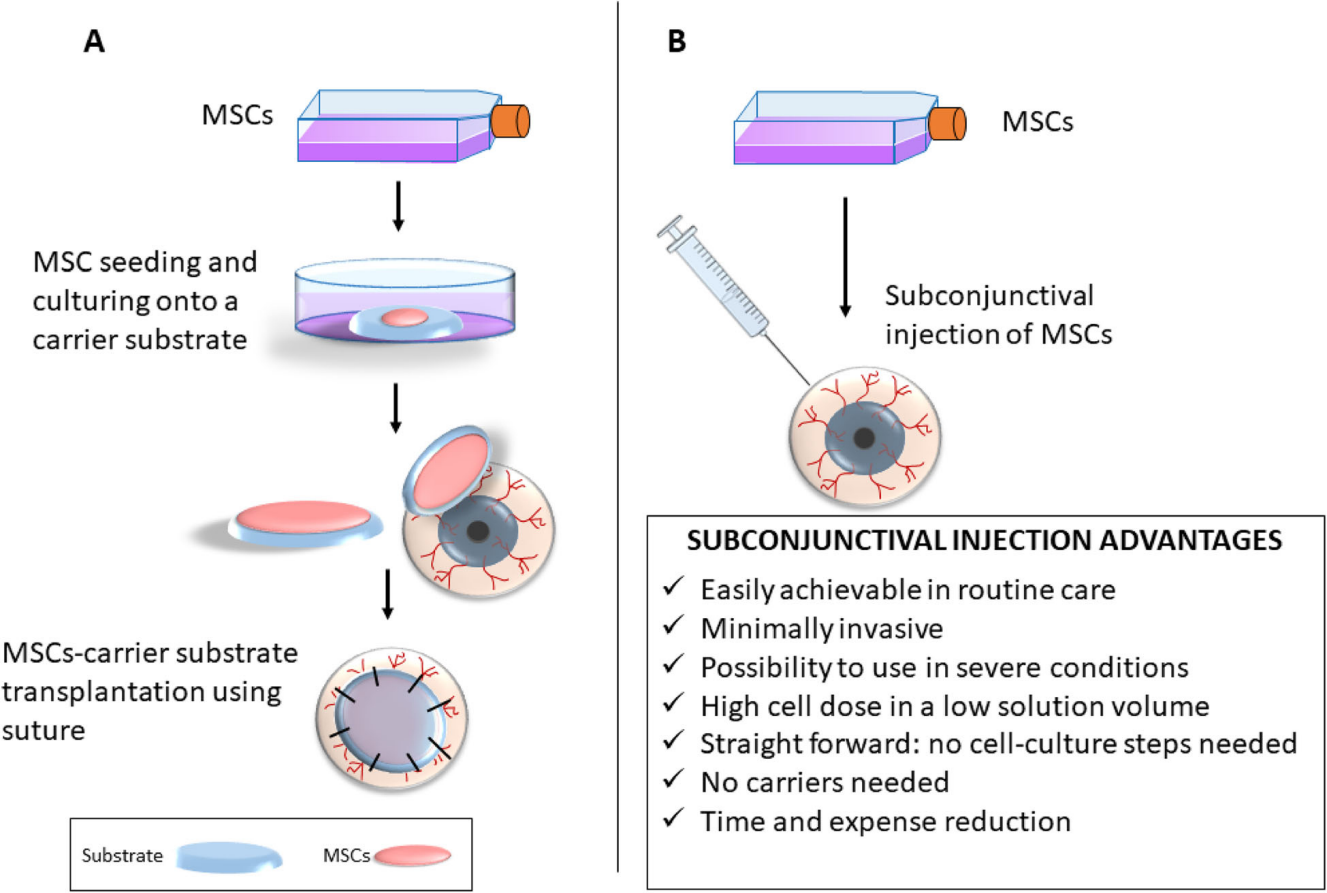
Comparison of MSC transplantation onto the ocular surface using a carrier substratum (**a**) versus subconjunctival injection (**b**). MSCs, mesenchymal stem cells

Considering all of the limitations associated with the classic cell transplantation routes, subconjunctival injection has clear advantages and has emerged as a viable alternative route for administering MSCs to the ocular surface. It is minimally invasive and easily achieved in routine clinical care for different treatments. It is normally indicated for the treatment of injuries in the cornea, sclera, anterior uvea, and vitreous. Clinicians regularly use subconjunctival injections of triamcinolone for macular edema [55], anti-microbial drugs for the treatment of infectious keratitis [56], mitomycin C in pterygium surgeries [57, 58], and bevacizumad for corneal neovascularization [59, 60] (Fig. 1). Moreover, this technique is described in several preclinical studies to treat different diseases such as uveitis, glaucoma, herpesvirus, inflammation, vascular hyperpermeability, edema, angiogenesis, retinoblastoma, choroidal neovascularization, and corneal grafts [13]. Moreover, it can be used in severe cases of LSCD, allows administration of high cell doses in a small volume, and does not need any extensive additional cell culture steps. Subconjunctival injections do not require the use of a surgical facility, and no additional post-injection interventions are required, resulting in a reduction of time and cost (Fig. 2).

### Subconjunctival MSC injection for treating corneal epithelial damage

In ophthalmology, subconjunctival injection is used to deliver drugs when the topical route is judged insufficient. This approach bypasses the epithelial cell barrier, ensuring rapid absorption. It can be used in severe conditions in which a high concentration of drug is needed [61]. Recently, subconjunctival injection has been used to administer MSCs in ocular surface therapy. Although multiple benefits have become evident, it is not easy to compare the published studies, as different animal models, methods, and cell doses and sources have been applied (Table 1).

**Table 1:** Subconjunctival injection of MSCs in experimental models of corneal epithelial damage

Species	Experimental model	Cell administration route	Follow-up time	Clinical signs	Cell migration	Anti-inflammatory/immunomodulatory effects	Corneal/limbal markers	Reference
Mouse	Corneal and limbal mechanical removal in diabetic mice	Mouse BM-MSCs: one injection (5 × 10^4^/5 μl PBS) immediately after damage	24, 48, and 72 h	↓Epithelial defect ↑Corneal epithelium proliferation	Migration to the limbal stroma and wound healing edge	↓Inflammatory infiltrates ↓CD45, CD86 ↓M1: TNFα, MCP-1 ↑M2: CD206, IL-10, Arg-1	↑P63 ↓K12	Di et al. [33]
	Corneal mechanical removal (2 mm trephine)	Mouse BM-MSCs: one injection (5 × 10^5^/10 μl PBS) 1 h after damage	2 and 4 days	↓Epithelial defect ↓Corneal opacity	Migration to the cornea and conjunctiva	↓CD45 ↓IL-1β, TNFα	–	Shukla et al. [62]
Rat	Corneal chemical burn (3 mm Ø disc/1 M NaOH 40s)	Rat BM-MSCs (2 injections 2 × 10^6^/100 μl PBS): one immediately, and one 3 days after damage	3–7 days	↓Neovascularization ↓Epithelial defect ↑Corneal epithelium regeneration	No migration. Cells located in the injection site	↓CD68 ↓MIP-1α, TNFα	–	Yao et al. [35]
	Corneal chemical burn (6 mm Ø disc/1 M NaOH 30s)	Rat BM-MSCs (2 injections 2 × 10^6^/100 μl PBS + polysaccharide hydrogel): one immediately and one 2 days after damage	3, 7, 14, and 28 days	↓Neovascularization (↓VEGF and ↑TSP-1) ↑Corneal epithelium regeneration ↓Corneal opacity	–	↓Inflammatory infiltrate ↑TGFβ ↓MIP-1α, TNFα	–	Ke et al. [36]
	Corneal chemical burn (4 mm Ø disc/1 M NaOH 30s)	Human limbal MSCs: one injection (2.4 × 10^6^/500 μl) 2 days after damage	1, 2, 3, and 4 weeks	↓Corneal opacity ↓Neovascularization ↓Epithelial defect	Migration to the corneal epithelium	↓Inflammatory infiltrate	–	Acar et al. [63]
	Corneal chemical burn (3 mm Ø disc/1 N NaOH 30s)	Rat BM-MSCs (1 × 10^6^/100 μl PBS): one injection 7 days after damage	7, 14, 21, and 28 days	↑Corneal wound healing ↓Neovascularization (↓VEGF and MMP-9) ↓Epithelial defect ↓Corneal opacity	No migration. Cells located in the injection site	↓Inflammatory infiltrate	–	Ghazaryan et al. [34]
	Corneal/limbal chemical burn (3 mm Ø disc/1 M NaOH 40s)	Rat BM-MSCs: one injection (2 × 10^6^/100 μl PBS) 3 days after damage	3, 6, 9, and 12 days	↓Corneal opacity ↓Neovascularization	–	–	–	Pan et al. [38]
	Corneal chemical burn (6 mm Ø disc 1 N NaOH 20s)	Rat BM-MSCs (pre-stimulated with TNF-α and non-stimulated): one injection (2 × 10^6^/100 μl PBS) immediately after damage	3, 7, and 14 days	↓Corneal opacity ↓Epithelial defect	No migration. Cells located in the injection site	↓Inflammatory infiltrates ↓CD68 ↓iNOS, TNFα, IL-1, IL-6, MCP-1, MIP-1α ↑PTGS2, TSG-6	–	Zhang et al. [64]
Rabbit	Corneal chemical burn (7 mm Ø disc/10% NaOH 40s)	Human AT-MSCs (1.3 × 10^5^/200 μl saline solution): one injection immediately after damage	30 days	↓Epithelial defect ↓Corneal opacity	–	–	↑Connexin-43 ↑β-catenin No changes in E-cadherin and p63	Lin et al. [37]
	Corneal chemical burn (6 mm Ø disc 1 N NaOH 30s on the upper cornea)	Combined administration: AT-MSCs (2 × 10^6^/500 μl) from rabbit by topical administration + stromal pocket + subconjunctival injection (immediately after injury)	3, 7, 14, 21, and 28 days	↓Corneal opacity ↓Neovascularization (↓VEGF) ↓Epithelial defect	–	↓Inflammation	–	Almaliotis et al. [65]
	Partial corneal/limbal chemical burn (4 mm Ø disc/1 M KOH 30s)	Human BM-MSCs or human limbal MSCs: one injection (5 × 10^3^/200 μl) immediately after damage	7, 14, 28 days, and 3 months	↓Corneal opacity ↓Neovascularization ↓Epithelial defect ↓Goblet cells in the cornea	Human limbal MSC: migration to the cornea	–	–	Li et al. [39]

Animal models of corneal epithelial damage in mice [33, 62], rats [34–36, 38, 63, 64], and rabbits [37, 65, 39] can be classified into two main groups. In one group, damage is restricted to the cornea [34–37, 62–64], whereas in the other group, both the cornea and the limbus are affected [33, 38, 39, 65].

The source of MSCs to be transplanted either allogeneically or xenogeneically is another variable, and BM, AT, and limbal MSCs are the most commonly used [33–39, 63]. For both allogeneic and xenogeneic transplantation, no immunosuppression was performed and no toxic or cell rejection reactions were reported. Therefore, subconjunctivally injected MSCs can be considered as a safe treatment in the different animal models so far reported and are currently being tested in a few clinical trials (see below).

Despite the advantages described above, subconjunctival injections also have five possible limitations, although some could be resolved with new studies. The first limitation is that there is still no consensus regarding the best cell vehicle solution. Second, it is necessary to establish a cell concentration in which the cells do not form clusters and consequently block the syringe during the injection. Third, it is not possible to inject a high volume of solution because it must be retained in and adsorbed by the conjunctiva. Fourth, there is yet no consensus regarding the number and location of the injections. The fifth limitation is that even though the technique is considered to be minimally invasive, it causes some pain in humans, and it could potentially allow an infection to occur.

To organize the information in the present review, we describe the main results regarding the therapeutic reduction of clinical signs such as corneal opacity and vascularization, as well as the anti-inflammatory and immunomodulatory effects. We also present results regarding cell migration and the corneal epithelial regeneration capacity after the subconjunctival MSC injection in experimental models of LSCD and/or corneal epithelial damage. Finally, we present a section that summarizes the clinical trials that are completed, have been planned, or are currently being performed using subconjunctival injections of MSCs for treating corneal epithelial damage in humans.

### Therapeutic effects of subconjunctival MSC injection following corneal epithelial damage

Several studies of corneal regeneration have reported the beneficial therapeutic effects of subconjunctival MSC injection. All the cited studies below used allogeneic cells unless specifically state otherwise. In diabetic mice, after mechanical removal of the corneal and limbal epithelium, subconjunctivally injected BM-MSCs decreased the epithelial defects and improved corneal reepithelization as confirmed by expression of Ki67 in the wound areas [33]. In another study, subconjunctival injection of BM-MSCs in mechanically damaged mice corneas reduced corneal opacity and epithelial defects [62]. Martinez-Carrasco et al., while not specifically studying a model of damage cornea, recently confirmed that the subconjunctival injection of human BM-MSCs in a mouse model of GVHD reduced the keratinization of the corneal epithelium mediated by PAX6 [66].

In addition, Yao et al. studied the effect of BM-MSC administration in a chemical burn model of rat corneas [35]. They applied two subconjunctival injections, immediately after the injury and again 3 days later. After 7 days, neovascularization was decreased as confirmed by the reduction of vascular endothelial growth factor (VEGF) expression, and the fast corneal epithelium recovery. Another study using a rat cornea burn model demonstrated that the use of a single subconjunctival injection of BM-MSCs was more efficient than AM BM-MSC transplantation [34]; however, the results were not strictly comparable because the number of cells transplanted by the AM was much lower. Nevertheless, the corneas getting subconjunctival BM-MSCs had greater decreases in epithelial defects, corneal opacity, and neovascularization associated with reduced vessel length and VEGF expression. Thus, the subconjunctival injection of BM-MSCs improved corneal wound healing during the 4-week follow-up more efficiently than the AM-transplanted BM-MSCs [34]. In a rat model of corneal alkali burn, the efficacy of subconjunctival BM-MSC injections combined with polysaccharide hydrogel treatment was investigated [36]. The reduction of epithelial defects, neovascularization, and corneal opacity were significantly enhanced by the combined treatment. Zhang et al. compared the subconjunctival injection of TNF-α–pre-stimulated BM-MSCs and non-stimulated BM-MSCs in a rat corneal burn model. In both cases, the epithelial defects were reduced. However, the corneal opacity decreased significantly only when TNF-α–pre-stimulated BM-MSCs were administered [64]. Interestingly, some researchers have also demonstrated that rat subconjunctival BM-MSC injections are effective in prolonging corneal allograft survival, reducing corneal opacity and neovascularization [67].

The administration of xenogeneic MSCs from human limbal stroma can reduce corneal opacity, neovascularization, and epithelial defects in alkali-burned corneas of rats [63] and rabbits [39]. Also, human limbal MSCs were more effective than human BM-MSCs in reducing the clinical signs [39]. Further, two corneal alkali burn models developed in rabbits have demonstrated that epithelial defects, corneal opacity, and neovascularization can be reduced by injecting a single dose of human AT-MSCs [37] or BM-MSCs [39]. Finally, in a partial LSCD model developed in rabbits, subconjunctival injection of AT-MSCs in combination with both topical application and injection into stromal pockets reduced the clinical signs of corneal opacity, neovascularization, and epithelial defects [65].

Clearly from the above considerations, it is not easy to directly compare the results from different works because each is performed under different conditions and protocols. However, it is interesting that, especially in rat models, even with different cell doses, different number of injections, and also different times of the injection, there is always improved corneal transparency, fewer epithelial defects, and decreased neovascularization. Therefore, to compare the outcomes more precisely, it is necessary to study the effects of MSCs derived from the same origin but applied in different doses and routes of applications in the same animal model. This approach will facilitate making comparisons and deciding which protocol gives the best results for that model, and perhaps provide insight regarding the application to human ocular surface disease.

### MSC migration after subconjunctival injection

MSCs can migrate to injured and inflamed areas through a mechanism that is mediated mainly by the chemokine CXCL12 that is produced in the damaged tissues and by the CXCR4 receptor present in the MSCs [68]. In diabetic mice with mechanical damage to the cornea and limbus, 2 days after subconjunctival administration of 5 × 10^4^ mouse BM-MSCs, Di et al. observed migration of the cells to the stroma of the corneal wound edge and also to the limbal stroma [33]. Consistent with these results, Shukla et al. also demonstrated the migration of mouse BM-MSCs to the corneal and conjunctival stroma 4 days after subconjunctival injection of 5 × 10^5^ cells in a mouse model of corneal mechanical injury [62]. Additionally, human limbal MSCs migrated from the limbus to the corneal epithelium 4 weeks after subconjunctival administration of 5 × 10^3^ and 2.4 x 10^6^ cells in rabbit and rat corneal burn models, respectively [39, 63].

However, not all studies have documented MSC migration from the injection site to the wound site. Four weeks after rat corneas received alkali burns and after subconjunctival administration of 1 × 10^6^ or 2 × 10^6^ rat BM-MSCs, Ghazaryan et al. [34], Yao et al. [35], and Zhang et al. [64] found no evidence of MSC migration to the corneas, demonstrating that the injected cells remained in the injection site. Additionally, human BM-MSCs showed no engraftment in the cornea of a mouse GVHD model 18 days after subconjunctival administration of 2 × 10^5^ human BM-MSCs [66]. However, the therapeutic effect of the MSCs was evident, indicating that the beneficial role of these cells is facilitated by trophic factors. It should be noted that in studies where no migration was observed, the MSCs were administered 3, 7, or 10 days after the creation of the damage [34, 35, 66]. In contrast, in most of the studies where the MSCs were injected on the same day that the injury was induced, migration to the limbus or cornea occurred [33, 62]. Thus, the delay in the administration of MSCs could induce a decrease in the migratory capacity of the cells due to a decrease in the signals released by the damaged tissues. Based on this hypothesis, it would be important to study how CXCL12 expression changes in the damaged tissues over time. The disparity in results is difficult to analyze because the studies used cells from different sources and species. A comparative study of CXCR4 expression in MSCs from different species and sources could provide insight regarding species-specific differences in MSC migration patterns.

### Anti-inflammatory and immunomodulatory effects of MSC subconjunctival injection in corneal epithelial damage

Several works have demonstrated the well-known anti-inflammatory effects of subconjunctivally injected MSCs [33, 34, 36, 63]. Investigations in mice demonstrated that subconjunctival injection of mouse-derived BM-MSCs produced a lower ocular surface inflammatory response in corneal mechanical damage models, preventing the infiltration of CD45-positive cells [62] and macrophages (CD86+) [33] into the cornea. Moreover, the secretion of some pro-inflammatory cytokines such as tumor necrosis factor (TNF)-α, interleukin (IL)-1β, and myocyte chemoattractant protein (MCP)-1 was reduced after subconjunctival injection of mouse BM-MSCs in these models [33, 62]. Additionally, Di et al. showed that TNF-α stimulated gene/protein (TSG)-6 combined with the MSCs, transformed the inflammatory monocytes into macrophages in the M2 state, limiting the immune response and expression of pro-inflammatory genes [33]. Furthermore, infiltration of T lymphocytes (CD3+) and expression of TNF-α were reduced in the ocular surface of a mouse GVHD model subconjunctivally treated with human BM-MSCs [66].

In different models of rat corneal burns, subconjunctival injections of rat BM-MSCs reduced infiltration of CD68+ macrophages and other inflammatory cells [34–36, 64]. Moreover, these studies agree on the decreased expression of pro-inflammatory cytokines such as TNF-α, IL-1, IL-6, and the chemotactic factors MIP-1α and MCP-1 in BM-MSC-treated rats [35, 36, 64]. Interestingly, Zhang et al. demonstrated that TNF-α–pre-stimulated BM-MSCs were more efficient at reducing inflammation than non-stimulated BM-MSCs [64]. Additionally, the increase in the expression of prostaglandin-endoperoxide synthase 2 and TSG-6 in the corneas treated with stimulated and non-stimulated BM-MSCs indicates that these molecules are implicated in the anti-inflammatory effect of the BM-MSCs [64]. Lu et al. confirmed that BM-MSC injection in a rat model of corneal allograft rejection decreased not only the CD68+ cells, but also the CD4+ T cells. At the molecular level, the anti-inflammatory action of this treatment was confirmed by (1) an increase in *Ptprc* gene expression, considered a CD45 antigen that regulates B and T cells, and (2) a reduction of *Hspa8* that is involved in inflammatory processes via MAPK [67].

Therefore, all of these works demonstrate that subconjunctivally injected MSCs reduce the infiltration of inflammatory cells into the cornea and decrease mainly TNF-α expression at the site of injury, promoting a less inflammatory microenvironment. Moreover, TSG-6 could be one of the molecules involved in the anti-inflammatory effect of the MSCs in the cornea.

### Expression of corneal/limbal epithelial markers after MSC subconjunctival injection

Analysis of corneal and limbal epithelial cell markers in the treated ocular surfaces is used to document the recovery of the specific cellular phenotypes after the subconjunctival injection of MSCs. In corneas of diabetic mice that were subconjunctivally injected with BM-MSCs, there was increased expression of the limbal epithelial stem cell marker p63 and decreased expression of the differentiated corneal epithelial cell marker K12 [33]. However, following alkali burn in rabbits, the expression of the corneal epithelial cell marker connexin 43 and the pro-proliferative marker β-catenin increased after AT-MSC injection [37]. In contrast, there were no differences in the corneal epithelial cell marker E-cadherin or in the limbal epithelial stem cell marker p63 expression after the treatment. To date, the role of the subconjunctivally administered MSCs in the recovery of the corneal and limbal phenotype is not clear yet. Consequently, this is an important field to further investigate.

### Subconjunctival injection of MSCs in clinical trials for treating corneal epithelial damage in humans

To date, five clinical trials appear in the database of the US National Institutes of Health ClinicalTrials.gov (Table 2), and to the best of our knowledge, no results have been published yet for any of these clinical trials. The first clinical trial performed by Boto et al. (Madrid, Spain) (*clinicaltrials.gov_NCT01808378*) was an interventional, phase 2, single-arm trial that has been completed according to Clinicaltrialsregister.eu (*clinicaltrialsregister.eu_2010-024328-53*). In this case, autologous AT-MSCs were used to treat total bilateral LSCD in 8 patients, applying 4 subconjunctival injections (4 × 10^6^ AT-MSCs per quadrant). Additionally, AMs were used, and 4 × 10^6^ AT-MSCs were topically dispensed to the damaged eye. The primary outcome in this trial was the feasibility and safety of autologous expanded lipoaspirated stem cells following 16 weeks of treatment for bilateral limbal-associated keratopathy. However, no results have been reported so far.

**Table 2:** Currently active clinical trials exploring subconjunctival injection of MSCs for treating corneal epithelial damages

**ClinicalTrials.gov** identifier / Clinicaltrialsregister.eu identifier	Condition or disease	Cell administration	Study design	Number of patients	Sponsor and performing center	Status and initiation date
NCT01808378 / EudraCT2010-024328-53	Keratopathy associated with bilateral LSCD	Human autologous AT-MSCs: 4 injections (4 × 10^6^ MSCs per quadrant) + topical application of 4 × 10^6^ MSCs for 20 min + amniotic membrane	Interventional, phase 2, single arm, unmasked	8	Research Institute of La Paz University Hospital, Madrid, Spain	Completed according to clinicaltrialsregister.eu 2012
NCT02325843	Corneal chemical burn	Human BM-MSCs: 1 injection of 5 × 10^6^ MSCs/500 μl + amniotic membrane. If persistent epithelial defect was noted, a second injection was performed.	Interventional, phase 2, single arm, unmasked	16	Sun Yat-sen University, Guangzhou, China	Completed 2014
NCT04484402	Corneal ulcer, corneal disease, corneal dystrophy	Autologous AT-MSCs + sodium hyaluronate 1% solution Autologous limbal stem cells + sodium hyaluronate 1% solution	Interventional, phase 1–2, three-arm parallel assignment, non-randomized, unmasked	25	Institute of Biophysics and Cell Engineering of National Academy of Sciences of Belarus	Completed 2016
NCT03237442	Corneal chemical burn	Human umbilical cord MSCs: 1 injection of 2 × 10^6^ MSCs/200 μl	Interventional, phase 1–2, two-arm parallel assignment, randomized, double masked	100	Guangzhou Saliai Stem Cell Science and Technology Co. Ltd., China	Not yet recruiting (unknown status) 2017
NCT03967275	Corneal chemical burn	Allogeneic human BM-MSCs	Observational, single arm	3	Ophthalmological Foundation Adolphe de Rothschild, Paris, France	Not yet recruiting 2019

Data from www.ClinicalTrials.gov and www.clinicaltrialsregister.eu

A new interventional, phase 1–2, three-arm parallel assignment, non-randomized, and unmasked clinical trial was carried out by Volotovsky et al. (Minsk, Belarus) and completed in 2019 (*clinicaltrials.gov_NCT04484402*). In this case, both autologous AT-MSCs and limbal stem cells were applied in 25 patients with inflammatory-dystrophic diseases of the cornea. Although no results have been published yet, treatment-related adverse effects and the number of cured patients were evaluated for 4 weeks and 2 months, respectively.

In addition, there are two more single-arm clinical trials in which subconjunctival injection of human BM-MSCs are being used to treat corneal chemical burns. The first one (*clinicaltrials.gov_NCT02325843*), under the direction of Dan et al. (Guangzhou, China), has been completed but no results have been reported so far. In it, one injection of 5 × 10^6^ human BM-MSCs was applied in 16 patients. This was followed by a second injection if a persistent epithelial defect was detected. The percentage of corneal perforations that occurred during a 3-month follow-up was analyzed, and different adverse events, such as ocular infection, conjunctival necrosis at the injection site, and retinal artery occlusion, as well as systemic complications during 6 months of follow-up, were also studied. The second single-arm clinical trial (*clinicaltrials.gov_NCT03967275*) by Gabison et al. (Paris, France) is still recruiting patients, and the dose of allogeneic human BM-MSCs is still unknown.

Finally, an as yet uninitiated interventional, phase 1–2, two-arm parallel assignment, randomized, and double-masked clinical trial (*clinicaltrials.gov_NCT03237442*) by Ma et al. (Guangdong, China) will compare the subconjunctival injection of 2 × 10^6^ human umbilical cord MSCs versus saline injection as potential treatment of corneal chemical burns.

## Conclusions

MSCs have several properties that make them a good choice for cell therapy in different tissues, including the cornea. The administration route is an important limiting factor for these treatments, as it should be safe and, when possible, minimally invasive. Subconjunctival injections are a minimally invasive and straightforward technique that is routinely used in ophthalmology to deliver drugs. Recent work has clearly shown that it can also deliver cell-based therapies, allowing the administration of higher cell doses. In addition, this technique could reduce costs as no substrata or surgical procedures are required.

Considering all the basic and translational investigations related to subconjunctival injection of MSCs for corneal regeneration, the convenience and interest of this technique is evident. Nevertheless, although the results of existing preclinical studies are very encouraging, to conclusively state that subconjunctival injections are a safe and effective route to administer MSCs to the ocular surface, it is necessary to carry out more of these studies. Additionally, the available clinical data from the ongoing clinical trials is still limited and insufficient; therefore, more clinical evidence is required to conclude if one route of administration is better than another in terms of clinical safety and efficacy. Nevertheless, considering the regenerative and anti-inflammatory effects shown by subconjunctival injection of MSCs in experimental models of corneal epithelial damage [33–39, 62–65] and the promising results obtained in the first clinical trial performed and published using BM-MSCs on AMs for treating patients suffering from LSCD [12], good efficacy would also be expected in LSCD patients when MSCs are administered by subconjunctival injections.

## Data Availability

Not applicable

## Abbreviations

AM: Amniotic membrane
AT: Adipose tissue
BM: Bone marrow
GVHD: Graft versus host disease
IL: Interleukin
LESC: Limbal epithelial stem cells
LSCD: Limbal stem cell deficiency
MCP: Monocyte chemoattractant protein
MHC: Major histocompatibility complex
MIP: Macrophage inflammatory protein
MSC: Mesenchymal stem cells
TGF: Transforming growth factor
TNF: Tumor necrosis factor
TSG: Tumor necrosis factor-α–stimulated gene/protein
TSP: Thrombospondin
VEGF: Vascular endothelial growth factor
